# Albumin Nanoparticles in Cancer Therapeutics: Clinical Status, Challenges, and Future Directions

**DOI:** 10.3390/pharmaceutics17101290

**Published:** 2025-10-02

**Authors:** Hachemi Kadri, Mesk Alshatfa, Feras Z. Alsalloum, Abdelbary Elhissi, Anis Daou, Mouhamad Khoder

**Affiliations:** 1 Drug Discovery, Delivery and Patient Care (DDDPC) Theme, School of Life Sciences, Pharmacy and Chemistry, Kingston University London, Kingston Upon Thames KT1 2EE, UK; k2121531@kingston.ac.uk (M.A.); feras.salloum98@gmail.com (F.Z.A.); m.khoder@kingston.ac.uk (M.K.); 2 Pharmaceutical Sciences Department, College of Pharmacy, QU Health, Qatar University, Doha P.O. Box 2713, Qatar; aelhissi@qu.edu.qa (A.E.); adaou@qu.edu.qa (A.D.)

**Keywords:** nanoparticles, protein, albumin, targeted drug delivery, cancer, clinical applications

## Abstract

Cancer, a global health burden, is characterized by uncontrolled cell growth and metastasis, often resulting in debilitating treatments and mortality. While conventional therapeutic strategies have improved survival rates, they are limited by challenges such as off-target toxicity and drug resistance. With their design to enable targeted drug delivery, nanoparticles have presented a promising avenue to overcome these limitations. Protein-based nanoparticles, particularly those based on albumin, are notable for their biocompatibility, stability, and ease of modification. The approval of Abraxane, an albumin-based nanoparticle formulation of paclitaxel, for metastatic breast cancer marked a significant milestone. However, further approvals have been slow to materialize until the recent approval of Fyarro^®^ in 2021. This focused review highlights the potential of albumin-based nanoparticles, emphasizing their advantages, current state, and progress in clinical use as anticancer therapeutics. We also discuss challenges impeding new approvals and future directions for unlocking the full potential of this technology.

## 1. Introduction

Cancer is a complex and multifaceted disease characterized by uncontrolled cell growth. Proliferating abnormal cells contiguously invade neighboring cells and can spread to distant organs through metastasis, which remains the leading cause of cancer-related mortality [1,2]. The transformation from normal to malignant cells is a multistep process, typically progressing from precancerous lesions to invasive tumors [3]. According to the International Agency for Research on Cancer (IARC), cancer is a leading cause of death globally, with approximately 10 million cancer-related deaths in 2022 [4]. Furthermore, the IARC projects that the global cancer burden will rise to 35 million new cases by 2050, representing a 77% increase compared to 2022 [5]. This emphasizes the urgent need for sustained efforts in research, treatment, and prevention to address the escalating global burden of the disease.

Conventional therapeutic strategies, which include surgery, radiotherapy, and chemotherapy, have improved cancer patient survival rates; however, their efficacy in achieving complete disease eradication is limited [6]. Surgical outcomes are often compromised by the invasive nature of malignant tissues and the risk of damage to adjacent organs [7]. Radiotherapy is associated with both acute and delayed adverse effects, such as skin reactions, fibrosis, and vascular or neuronal injury [8]. Chemotherapeutic agents, when delivered via traditional systems, lack specificity for cancer cells, resulting in severe adverse effects, including dose-dependent hematotoxicity, cardiotoxicity, and the development of multidrug resistance with prolonged exposure [9,10,11]. Tumor heterogeneity further complicates treatment, as the evolving molecular landscape of cancer leads to diverse cell populations with variable therapeutic responses [12]. Additionally, the traditional delivery of anticancer agents suffers from other drawbacks like poor drug solubility, limited bioavailability, and pre- or post-absorption inactivation.

To address these challenges, there is a pressing need for unconventional targeted delivery strategies. Nanoparticulate drug delivery systems offer the potential to selectively target cancer cells while minimizing damage to healthy tissues, thereby improving therapeutic efficacy and reducing side effects [13]. Owing to their versatility and tunable properties, nanoparticles (NPs) can improve drug stability and bioavailability, accelerating the development of more effective cancer therapies [14]. To date, several classes of NPs have been developed and investigated for targeted cancer drug delivery. Of these classes, protein nanoparticle (PNPs), namely albumin-based NPs, gained more attention, especially after nab-paclitaxel (Abraxane^®^) received FDA approval for metastatic breast cancer in 2005 [14].

This review contextualizes the significance of protein-based NPs with a focus on albumin-based formulations, their clinical applications in cancer treatment, the challenges they encounter, and future possibilities for advancing this technology.

## 2. Albumin Nanoparticles: Properties and Methods of Preparation

**Figure 1:**
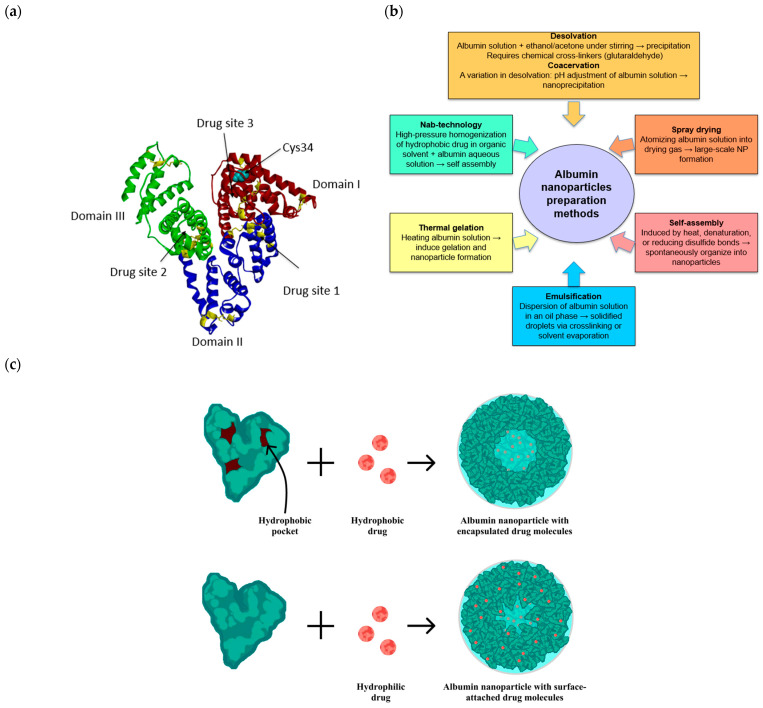
(**a**) Three-dimensional structure of human serum albumin (HSA), illustrating its three homologous domains shown in red (I), blue (II), and green (III), and drug sites. Disulfide bridges are depicted as yellow sticks, and cyan spheres highlight the available cysteine 34 in domain I. The 3D model was generated by Biovia Discovery Studio (https://www.3ds.com/products/biovia/discovery-studio, accessed on 3 June 2025) using the 1AO6 PDB file. (**b**) Schematic overview of major albumin NP preparation methods. (**c**) Schematic representation of an albumin nanoparticle illustrating hydrophobic drug encapsulation and hydrophilic drug surface attachment, adapted from reference [23].

PNPs represent a highly versatile drug delivery system for anticancer therapy due to their structural and functional properties. While the most used proteins in NP formulation are gelatine and albumin, the unique characteristics of albumin have significantly contributed to its widespread adoption in various medical applications [15]. Albumin, the most abundant protein in human plasma, is notable for its resistance to heat, pH fluctuations (typically between 4 and 9), and many organic solvents [16,17]. Furthermore, the three-dimensional structure of human serum albumin (HSA) (Figure 1a), comprising three homologous domains with multiple hydrophobic pockets and abundant drug-binding sites, enables efficient encapsulation of diverse bioactive agents, thereby reducing required drug amounts and enhancing tumor cell targeting [18]. The three major drug-binding pockets are located within domains I, II, and III (Figure 1a). These hydrophobic cavities, shaped by surrounding polar and basic amino acid residues, govern drug selectivity: the site in domain II binds bulky heteroaromatic drugs (e.g., warfarin, phenylbutazone, indomethacin), the site in domain III accommodates smaller aromatic and neutral hydrophobic drugs (e.g., ibuprofen, benzodiazepines, propofol), while the site in domain I preferentially recognizes endogenous ligands such as bilirubin, heme, and fatty acids, along with certain drugs (e.g., naproxen, lidocaine) [19,20].

Albumin can be obtained from different sources. While egg albumin (also known as ovalbumin) is widely used in the food industry, bovine serum albumin (BSA) and HSA, known for their biocompatibility, have received significant attention in the pharmaceutical field, particularly for NP formation [18]. Although BSA and HSA share about 76% sequence identity and highly conserved three-domain architectures with similar ligand-binding pockets [21], BSA is xenogeneic and can elicit immune responses, whereas HSA is endogenous and generally non-immunogenic in humans, making it the safer choice for clinical applications [22,23]. Albumin-based NPs exhibit an extraordinary capacity at accommodating both hydrophobic and hydrophilic therapeutic agents, whether unionized, positively or negatively charged. This adaptability stems from the abundance of the various functional groups derived from amino acids (e.g., carboxyl, amino, thiol, and hydroxyl groups). These groups provide multiple interaction mechanisms—including hydrogen bonding, electrostatic interactions, hydrophobic forces, and covalent conjugation—that facilitate the binding or encapsulation of drugs with a wide spectrum of physicochemical properties, hence expanding the range of potential therapeutic applications [18]. Additionally, these functional groups enable surface modification of albumin NPs, allowing for the conjugation of targeting ligands to enhance specificity for targeted drug delivery [24]. Notably, the free thiol at Cys34, the only unpaired cysteine residue in HSA, provides a unique reactive site for covalent drug conjugation and further functionalization, expanding the versatility of albumin-based nanocarriers [18]. Figure 1c illustrates hydrophobic and hydrophilic drug loading.

Albumin NPs can be prepared by several techniques, each distinct in its approach. These methods have been comprehensively reviewed by Elzoghby et al. [25] and summarized in Figure 1b. The desolvation method is commonly used, whereby a dehydrating agent, such as ethanol or acetone, is added to the albumin solution under continuous stirring. This process exposes the albumin hydrophobic region to water, reducing its solubility, thereby leading to the precipitation of albumin NPs. Chemical crosslinkers, often glutaraldehyde, are used to stabilize the NPs. The coacervation technique is a variation in desolvation where the pH is used to adjust the albumin water solubility, enabling the desolvation agents to induce the nanoprecipitation. Self-assembly is another approach in which albumin molecules spontaneously organize into nanoparticles, a process that can be induced by heat, denaturation, or chemical reduction of internal disulfide bonds. The emulsification technique involves dispersing an albumin solution in water-immiscible oil, followed by solidification of the albumin droplets through chemical crosslinking to form NPs. Thermal gelation, inducing NP formation via temperature changes, allows for NP production under mild conditions. For the large-scale production of albumin NPs, the nano-spray-drying method can be used. In this technique, albumin solution is atomized through a nozzle to form a spray that dries through a gas and forms albumin NPs.

Nanoparticle albumin-bound (nab) technology is an innovative method for the encapsulation of hydrophobic drugs into albumin NPs. The first nanoparticle-based product approved and marketed was produced by Nab-technology. In this process, a hydrophobic drug is dissolved in an organic solvent, and that is emulsified in an albumin aqueous solution. This primary oil in water emulsion is then subjected to high-pressure homogenization, leading to the albumin self-crosslinking and the formation of an albumin layer around the drug core. Finally, the solvent is evaporated, yielding stable albumin NPs [26].

## 3. Clinical Applications in Cancer Treatment

**Figure 2:**
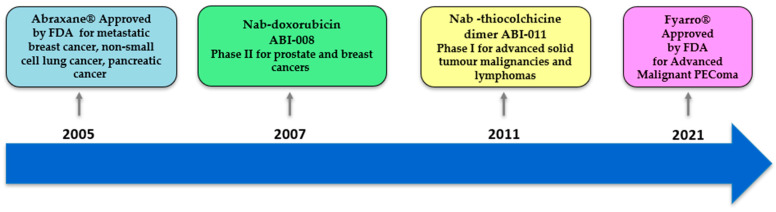
Timeline of clinical milestones of albumin-based nanoparticle anticancer therapeutics.

Albumin-based NPs have demonstrated significant potential in delivering anticancer drugs to tumor sites, enhancing drug accumulation and overcoming drug resistance [27]. To date, four albumin-based nanomedicines, all of which are prepared by Nab-technology, have been tested in clinical trials for potential use as cancer therapeutics. Abraxane^®^ (ABI-007), also known as nab-paclitaxel, represents a significant milestone as the first albumin-based drug approved for the treatment of metastatic breast cancer by the FDA in 2005 [14,28]. Its indications have since expanded to include non-small cell lung cancer and metastatic pancreatic cancer [14]. Abraxane^®^ takes advantage of albumin’s natural properties to enhance the delivery of paclitaxel, which is otherwise hindered by its poor water solubility, facilitating higher dosage administrations within shorter periods. Despite some dose-limiting adverse effects, clinical trials have shown that Abraxane^®^ exhibits superior performance compared to traditional paclitaxel treatments, evidenced by higher response rates, longer times to tumor progression, and increased median survival rates in breast cancer patients [28]. Abraxane^®^ also demonstrated significant therapeutic effects in combination with other treatments, such as trastuzumab, bevacizumab, carboplatin, 5-fluorouracil, and gemcitabine [29]. Fyarro^®^ (nab™ rapamycin), an mTOR inhibitor, is another Nab-technology albumin nanoparticle that was approved by the FDA in 2021 for advanced malignant perivascular epithelioid cell tumor (PEComa) in adults [30]. Other anticancer-loaded Nab-technology albumin NPs that entered clinical trials include ABI-008 (nab-docetaxel) and ABI-011 (nab-thiocolchicine dimer) [29,31]. The clinical application and stage of these therapeutic agents are summarized in Table 1, and their chronological milestones are illustrated in Figure 2.

## 4. Challenges

The use of Albumin NPs has attracted considerable attention, especially with the approval of Abraxane^®^. However, several challenges need to be addressed to maximize their application and effectiveness in clinical settings. While the biocompatibility and low immunogenicity nature of albumin are significant advantages, the conjugation or encapsulation of therapeutic agents can induce protein conformational changes, potentially increasing immunogenicity [33]. Although albumin NPs are inherently robust under biological conditions, crosslinkers are often required to ensure sustained stability and controlled drug release [34]. The toxicity associated with traditional crosslinkers, like glutaraldehyde, along with their potential to interfere with drug efficacy and induce protein denaturation, has prompted the exploration of natural crosslinkers such as glucose and tannic acid [35,36]. For instance, glucose-crosslinked BSA nanoparticles by Amighi et al. showed comparable physicochemical characteristics (e.g., 125.1 nm vs. 76 nm size, similar zeta potential, and FTIR) to glutaraldehyde-crosslinked counterparts, supporting glucose as a less-toxic option [36]. Further, glucose under UV irradiation yielded albumin nanoparticles with comparable size, cellular uptake, and biphasic drug release compared to glutaraldehyde systems, but with reduced cytotoxicity [37]. Tannic acid has also proven effective, forming protein interactions that, when combined with natural genipin (a biocompatible crosslinker), improved nanoparticle stability, delayed gastric release, and enhanced in vivo efficacy [38]. This trend towards eco-friendly crosslinkers for stable, low-toxicity albumin NPs is actively promoted in recent research [39]. These natural alternatives offer a promising route to refining nanoparticle production processes for optimal stability, size, and safety.

The synthesis of albumin NPs also presents a set of challenges. Preparation methods have various inherent drawbacks, ranging from the risk of mechanical shear force-induced destruction in emulsification and double emulsification techniques to the necessity for toxic chemical crosslinkers in desolvation and pH coacervation methods [25]. Despite minimizing surfactant use, nab technology requires the use of toxic organic solvents like chloroform and dichloromethane to dissolve hydrophobic drugs, introducing potential risks of residual toxicity and environmental damage [25,26]. Other synthesis methods, such as thermal gelation and spray drying, are limited by issues related to drug heat-sensitivity and potential protein denaturation [26].

The production of albumin-based formulations is both complex and costly, driven by the need for high-purity albumin, which raises product costs and may limit accessibility. Additionally, the inherent batch-to-batch variability in natural polymers, like albumin, requires strict control over the characteristics of NPs to ensure safety and efficacy. Scaling up production and regulatory compliance present further challenges, often requiring process modifications or new manufacturing approaches.

## 5. Future Directions for Albumin Nanoparticles in Cancer Treatment

Albumin-based nanosystems are experiencing a resurgence propelled by advances in nanomaterials and drug delivery methodologies. Recent studies have introduced promising approaches to enhance the targeting ability, cellular internalization, and drug accumulation of albumin NPs. For example, modification of albumin NPs with long-chain fatty acids, like C18, has been shown to improve encapsulation efficiency of hydrophobic drugs such as doxorubicin, resulting in higher drug loading, controlled release, enhanced stability, reduced side effects, and improved anticancer activity [40]. Another successful modification involves incorporating 4-carboxyphenylboronic acid (CPBA), a biocompatible ligand for drug-targeted delivery. CPBA interacts with overexpressed sialic acid on cancer cells, enhancing the uptake of the sorafenib and simvastatin coloaded NPs [41].

Innovative conjugation methods to modify albumin NPs through diverse chemical reactions have recently attracted considerable attention. For example, the Maillard reaction was utilized to attach maltose to bovine serum albumin. This method offered a straightforward approach, given the simplicity and well-known nature of the Maillard reaction between reducing sugars and amino groups in proteins. This approach led to reduced systemic toxicity of doxorubicin and improved stability of the resulting NPs [42].

Integration of immunotherapy and chemotherapy is another emerging direction that utilizes albumin NPs to enhance immune responses against cancer cells. For instance, a novel nanotherapeutic agent (PDL1-NP-FEXO), created by attaching PD-L1 aptamers to albumin nanoparticles loaded with the H1-antihistamine fexofenadine (FEXO), demonstrated higher antitumor activity, suggesting that this combination strategy may enhance the effectiveness of checkpoint blockade (ICB) therapy [43]. Building on this, dual-functionalized albumin NPs with both PD-1 and PD-L1 aptamers have been designed to simultaneously target immune and tumor cells, amplifying antitumor immune responses [44]. Beyond immunotherapy, albumin NPs have also been exploited for targeted small-molecule delivery. Protocatechuic acid-encapsulated albumin nanoparticles conjugated with folic acid enabled folate receptor-mediated targeting, which increased selective uptake into cancer cells and thereby amplified anticancer efficacy while reducing toxicity to normal tissues [45]. Folic acid-conjugated albumin NPs have also been engineered as pH-responsive carriers that exploit the acidic tumor microenvironment. Some systems further incorporate GSH responsiveness for dual redox- and pH-triggered release, while others are co-loaded with baicalin to synergize with tamoxifen, improving therapeutic efficacy in estrogen receptor α–positive breast cancer [46,47].

More recently, albumin NPs conjugated with azocalix[4]arene have gained hypoxia-responsive drug release capacity. When co-loaded with hydroxychloroquine and an oxygen-independent photosensitizer, these NPs achieved sequential mitophagy modulation and amplified oxidative stress, resulting in potent cytotoxicity in hypoxic tumors [48].

## 6. Conclusions

In conclusion, albumin nanoparticles present a highly versatile platform for delivering anticancer agents with enhanced efficacy and reduced systemic toxicity. The clinical success of Abraxane^®^ has demonstrated the feasibility and benefits of albumin-based nanomedicines and paved the way for further exploration and development in this field. Nevertheless, the translation of additional albumin NPs into approved clinical therapies has been slow and hampered by challenges related to stability, synthesis, and production costs. Overcoming these obstacles by fine-tuning their drug targeting and release mechanisms, along with the adoption of innovative biorthogonal fabrication technologies, is essential for unlocking their full potential in cancer therapeutics and facilitating future approvals. Moreover, their success in cancer treatment could enable applications in other medical conditions.

## Figures and Tables

**Table 1:** Albumin-based NPs that entered the clinic as an anticancer therapeutic.

Treatment	Type	Clinical Stage	Clinical Application	Refs.
Abraxane^®^ Nabpaclitaxel (ABI-007)	HSA-bound paclitaxel NP	Approved by FDA	Metastatic breast cancer, non-small cell lung cancer, pancreatic cancer	[28,32]
Fyarro^®^ Nab-rapamycin (ABI-009)	Albumin-bound rapamycin NP	Approved by FDA	Advanced malignant PEComa in adults	[30]
Nab-docetaxel (ABI-008)	Albumin-bound docetaxel NP	Phase I/II *	Prostate and breast cancers	[31]
Nab-thiocolchicine dimer (ABI-011)	Albumin-bound thiocolchicine dimer	Phase I *	Advanced solid tumor malignancies and lymphomas	[29]

* Note: According to www.clinicaltrials.gov, the available trials for these agents (ABI-008, ABI-011) are listed as terminated or completed, and no further clinical development is ongoing as of 2025.

## Data Availability

Data sharing is not applicable. No new data were created or analyzed in this study.
